# The Relationship Between Stress, Academic Motivation, and Subjective Vitality Among Nursing Students

**DOI:** 10.3390/nursrep15080300

**Published:** 2025-08-15

**Authors:** Stanislav Sabaliauskas, Kamile Ingelevič, Oksana Misiūnienė, Agnė Jakavonytė-Akstinienė

**Affiliations:** Department of Nursing, Faculty of Medicine, Institute of Health Science, Vilnius University, M. K. Čiurlionio St. 21, LT-03101 Vilnius, Lithuaniaoksana.misiuniene@mf.vu.lt (O.M.); agne.jakavonyte-akstiniene@mf.vu.lt (A.J.-A.)

**Keywords:** nursing students, stress, learning motivation, vitality

## Abstract

**Objectives**: This cross-sectional, descriptive correlational research investigated the relationship between stress, academic motivation, and subjective vitality among nursing students. **Methods**: Participants were recruited through a non-probability purposive sampling approach. An anonymous online survey was conducted with 188 first- to fourth-year study nursing students, assessing their perceived academic stress using the Perceptions of Academic Stress Scale, academic motivation using the Student Academic Motivation Scale (SAMS-21), and subjective vitality using the Subjective Vitality Scale. Descriptive statistics, bivariate correlational analysis, and multivariate analysis were employed in this study. **Results**: The results indicate that students experience moderate stress levels during exam sessions, with higher stress associated with workload and examinations. Academic motivation was characterized by high extrinsic motivation, which identified regulation and intrinsic motivation to know. A significant difference in a form of extrinsic motivation—introjected regulation—was found between student groups, with a tendency for this motivation to decrease over the years of study. No statistically significant relationship was found between students’ academic stress and subjective vitality. **Conclusions**: Academic stress related to workload and exams is determined by both demographic factors, such as age and year of study, and psychological factors, including academic self-perception and amotivation, which highlight the multifaceted nature of the stress experienced by nursing students. Students’ subjective vitality is related to intrinsic motivation—to know and achieve—and to all extrinsic motivation. External forms of regulation, especially introjected regulation, are significantly related to students’ subjective vitality.

## 1. Introduction

The study period is one of the key stages in developing a conscious personality. Students take responsibility for their education to become qualified professionals during their studies. However, the study period presents different challenges and stresses for students. Coping with stress depends on recognizing stress reactions and perceiving emotional responses to stress [1,2]. Research has shown that nursing students experience higher stress levels than students from other specialties [3].

High academic demands, frequent exams, students’ disorganization, and inability to manage their time effectively and devote sufficient time to studying and preparing for exams can lead to increased stress, which in turn can inhibit cognitive function, reduce understanding and interest in studying, and promote a lack of energy [4,5]. These processes can directly affect students’ concentration and memory, reduce their motivation to learn, and hinder the learning process and their exam success. Undoubtedly, motivation to learn is an integral factor in successful learning. However, stress can hurt learning motivation [6,7], emotions, and study performance [7].

Although there is a large body of research in the global literature on the effects of stress on students’ motivation to learn, emotional state, and academic performance [7], there is still a lack of studies in the Lithuanian context that provide a comprehensive analysis.

### 1.1. Student Stress in an Academic Environment

According to the World Health Organization, stress is a natural, threat-oriented response that depends on an individual’s ability to perceive and manage complex situations [8]. Academic stress is associated with learners’ reactions to changing external and internal demands and manifests as psychological tension arising from attempts to adapt to educational challenges. If the level of stress experienced is in line with an individual’s adaptive capacity, stress can act as a mobilizing resource, increasing achievement motivation and enhancing emotional resilience. However, beyond individual tolerance limits, stress becomes a destructive phenomenon that disturbs an individual’s mental equilibrium and impairs their ability to perform tasks constructively [9].

In academic contexts, stress is conceptualized as a multidimensional phenomenon involving cognitive, emotional, and behavioral components [5,10,11,12,13]. Cognitively, students may experience reduced concentration, impaired decision-making, distractibility, and heightened sensitivity to errors or criticism. Emotionally, stress often manifests as anxiety, fear, and tension, while behaviorally, it can lead to maladaptive coping, such as sleep disturbances or substance use. These symptoms interfere with the students’ psychological functioning and overall academic engagement.

The stress experienced by students stems from a range of external academic demands and psychological factors. Academic stressors include exams, thesis presentations, time pressure, and formal assessment requirements [1,14,15,16,17]. Subjective stressors, such as fear of failure, fluctuating self-esteem, and perceived academic competition, further intensify emotional burden. Additionally, individual characteristics such as time management skills, availability of social support, and emotional regulation play a crucial role in determining how students experience and respond to academic stress [5,18,19].

### 1.2. Academic Motivation of Student Nurses

Motivation is key to student engagement in academic activities, academic achievement, and professional preparation. Research shows that students generally have a moderate to high level of academic motivation [20,21,22]. Nevertheless, individual studies have found low motivation levels among nursing students [23], which is explained by the fact that cultural, academic, and organizational environment characteristics can strongly influence motivation.

Research confirms that high motivation for learning leads to higher student effort and better learning outcomes and is also associated with positive emotions. Motivated students are more proactive in finding effective strategies to overcome challenges [6]. However, motivated students are more likely to be stressed and less satisfied with their studies [24].

It has been observed that upper-year students with more substantial professional commitment have higher motivation to study [25]. However, adverse academic outcomes, such as failing exams, can reduce learning motivation and even lead to dropping out [26]. In contrast, studies conducted with nursing and paramedic students have revealed the interesting fact that students with high levels of motivation to learn may be at risk for burnout in the long term.

Additionally, the stress of being under constant pressure directly impacts future nurses’ motivation to learn. Research shows that while moderate stress levels can promote academic motivation, high or prolonged stress decreases motivation, impairs concentration, weakens critical thinking, and impairs academic achievement, enthusiasm for learning, self-efficacy, and resilience [6,16,27,28,29]. Inadequate teacher support, high learning demands, and fear of making mistakes are particularly detrimental [27]. These trends underline the need to strengthen psychological support measures in the academic environment to maintain students’ motivation and well-being during intensive study.

### 1.3. Subjective Vitality in Students’ Academic Environment

Vitality is a dynamic aspect of well-being that expresses a subjective sense of energy, aliveness, and the ability of an individual to function actively at both physical and psychological levels [30,31]. In a broader sense, vitality refers to the presence of energy and a person’s inner sense of ‘being alive’, associated with emotional upliftment, enthusiasm, and social activity [32]. In the context of well-being, vitality refers to good physical health and the absence of fatigue or disease at the somatic level. From a psychological perspective, it relates to self-integration, efficiency, and strength [33]. Therefore, vitality is an essential internal resource that influences psychological adjustment and academic achievement.

High vitality is generally associated with greater stamina, initiative, and mental and physical strength [34]. Although research evidence typically suggests that physical activity, such as active sports, is positively correlated with increased subjective vitality levels [35,36], the academic environment, as a significant social context, can strongly influence students’ psychological well-being and vitality [37]. Stress and psychological strain reduce feelings of vitality [32]. An empirical study of students revealed that the level of anxiety before a stressor predicts changes in vitality, whereas cortisol levels are not significantly associated with a decrease in vitality [38]. These findings underline that subjective emotional processes may affect viability more than physiological stress indicators.

The academic performance of students is closely related to their motivation and achievement. Research shows that subjective vitality depends not only on physical health but also on the strength of intrinsic motivation [39]. Higher levels of intrinsic motivation, manifested by a desire for self-directed learning and development, support and enhance vitality [40,41]. Vitality also helps students overcome academic challenges such as workload, failure, or complex assignments, encouraging them to maintain sustained engagement and resilience to stress.

#### The Present Study

The stress experienced by students arises from a complex interaction of academic, psychological, social, and economic factors. The most common stressors are related to excessive study load, lack of sleep and rest, difficulties in time management, low grades, parental expectations, and a competitive environment during studies [15,17,18,19]. Individual factors, such as fear of failure, lack of self-confidence, and emotional experiences formed before studies, are also important. These interrelated factors can reduce academic motivation, weaken subjective vitality, and negatively affect the learning outcomes. Therefore, identifying sources of academic stress and systematically assessing them are essential for creating a psychologically safe and motivating study environment.

Research findings show that student stress is widespread and often exceeds the thresholds considered conducive to health [3,16,42,43,44,45]. Although prevalence rates vary depending on measurement methods and sociocultural contexts, general trends indicate that a significant proportion of students experience moderate to high levels of stress, which negatively affect their psychological well-being, academic achievement, and professional preparedness [46]. Among higher education populations, nursing students are particularly vulnerable due to the intensive demands of integrating theoretical knowledge with clinical practice. The combination of high academic expectations, constant evaluations, and the emotional weight of professional responsibility places these students under considerable psychological strain, which—if left unmanaged—can contribute not only to learning difficulties but also to longer-term mental health problems [47].

Academic stress has been shown to significantly affect students’ learning effectiveness and cognitive performance. Elevated stress levels are linked to impairments in attention, working memory, and executive functions, all of which are critical for effective learning [48]. Additionally, stress negatively influences higher-order cognitive processes such as critical thinking, creativity, and problem-solving abilities [49]. Students experiencing chronic academic stress may also struggle to demonstrate their full knowledge potential, particularly in high-pressure situations, such as exams or public presentations [50]. Thus, stress reduces a person’s potential to deal effectively with tasks or situations.

Nursing students often face multiple stressors, such as high workload, clinical responsibilities, and performance pressures, which can compromise their academic engagement and psychological well-being [51]. Research has shown that high levels of academic stress are associated with reduced intrinsic and extrinsic motivation [52], which is often associated with reduced vitality, a subjective state characterized by energy, vitality, and mental resilience [53]. Therefore, stressful situations can have a negative impact on effective problem-solving. However, understanding the interactions between these factors is essential for creating a supportive learning environment that promotes not only academic achievement but also students’ emotional and psychological functioning.

Students’ stress, motivation, and subjective vitality in the academic environment directly impact their psychological well-being and academic performance. However, the interaction between these factors is still not well understood. Existing research tends to analyze stress, motivation, or vitality in isolation, and there is a lack of systematic knowledge about their complex relationships during academic studies. The aim of this study is to explore the relationship between stress, academic motivation, and vitality experienced by nursing students during a session. The following hypotheses are proposed in this study:

**H1.**  *Students report the highest levels of academic stress in relation to workload and examinations.*

**H2.**  *There is a significant association between students’ academic motivation and their subjective vitality.*

**H3.**  *There is a significant negative association between students’ subjective vitality and their perceived academic stress.*

## 2. Materials and Methods

### 2.1. Study Population and Participants

The study population consisted of 358 nursing students. To ensure the reliability of the results, a 95% confidence level and a 5% margin of error were chosen. Based on these parameters, a sample size of 186 respondents is considered adequate. Such a sample size allows for the collection of statistically reliable data that can be generalized to the entire study population with an acceptable level of error [54,55]. The open access program G*Power, version 3.1.9.2, was used to calculate the study sample.

A non-probability purposive sampling method was used to recruit the participants. A total of 188 students of the nursing study program at the Faculty of Medicine of Vilnius University, 1st–4th year of study, participated (Table 1). The students ranged from 18 to 27 years, with a mean age of 20.75 (median 21 years). Study participants were 98.9% women (*n* = 186) and 1.1% men (*n* = 2).

### 2.2. Instruments

The introductory part of the questionnaire explained the purpose of the study and provided information regarding the ethical framework. The sociodemographic questions included information on the participants’ gender, age, and course of study.

The Perceptions of Academic Stress Scale [56] assessed perceived academic stress and its sources. The scale consists of 18 statements divided into three subscales: academic expectations, workload and examinations, and academic self-perceptions. The answers were rated on a 5-point Likert scale, with lower scores (mean = 1) representing the highest levels of stress and higher scores (mean = 5) representing the lowest levels of stress. The results indicated that respondents, on average, reported a moderate level of stress, corresponding to a mean score of 3. The scale demonstrated internal consistency, confirming its reliability for data analysis (Cronbach’s alpha = 0.750).

The Student Academic Motivation Scale (SAMS-21) was used to measure students’ motivation. This scale is based on a hierarchical model of motivation [57,58,59]. The SAMS-21 is subdivided into seven subscales, which measure three types of intrinsic motivation (intrinsic motivation to know, accomplish things, and experience stimulation), three types of extrinsic motivation (extrinsic, introjected, and identified regulation), and amotivation. Participants rated the statements on a 7-point Likert scale. The scale demonstrated good internal consistency, confirming its reliability (Cronbach’s alpha = 0.950). Autonomous and controlled motivation were calculated by performing regression analysis [60].

The Subjective Vitality Scale is based on the self-determination theory [61] and is designed to assess students’ subjective vitality and energy. The scale consists of seven statements rated by the participants on a 7-point Likert scale. The scale demonstrated good internal consistency, confirming its reliability for data analysis (Cronbach’s alpha = 0.921).

### 2.3. Survey Design and Procedure

The participants were enrolled in this cross-sectional, descriptive correlational research using a snowball sampling method [62]. An anonymous online survey was conducted to ensure the participants’ anonymity and confidentiality to achieve the study’s aim. Data collection was performed remotely. Links to the questionnaire were sent to the class representatives of the 1st- to 4th-year nursing study program at the Faculty of Medicine of X University with a request to share the link with their classmates. Data collection was carried out until a representative number of participants was reached.

The authors of the Perceptions of Academic Stress Scale did not specify a specific period when it was appropriate to apply the scale. However, the scale is particularly suitable for use during periods when students experience increased academic workload and stress, such as during intensive assessments or exams, or during the adaptation period. This allows for the assessment of stress levels when they affect students’ psychological well-being and motivation to learn the most. Obviously, the highest stress level could be determined on the day of the exam, but such a request from students would be an additional concern on the day of the exam and would significantly reduce their willingness to participate in the study and answer questions. Therefore, a period of three days before the exam in subject X was chosen, when students were already intensively preparing for the exam. The link to the questionnaire was shared during the winter exam session from December 2023 to January 2024.

### 2.4. Data Analysis

Descriptive statistics were used to present the data meaningfully, and regular distribution testing was performed on the variables. The chi-square (χ^2^) test was used to analyze statistical relationships. Spearman’s correlation (r) method assessed the relationships between variables. The reliability or internal consistency of the questionnaires was assessed by calculating Cronbach’s α. Hierarchical regression analysis was used to explain the relationship between one scale dependent variable and independent variables. Statistical significance was defined as *p* < 0.05 for all tests. Statistical analyses were performed using the IBM SPSS Statistics software (version 29; IBM Corp., Armonk, NY, USA).

### 2.5. Research Ethics

The principles of anonymity, confidentiality, and respect for participants were followed in conducting the study. Participants were provided with full information about the study and assured that the information provided by the participants would not reveal their identity and would only be used for the study [63]. All subjects participated voluntarily, and informed consent was obtained from all participants. All the participants were informed of the possibility of withdrawing their participation at any time. The participants agreed to participate in the study by completing an online questionnaire. The Declaration of Helsinki was followed in the study, and the study protocol was approved by the Research Ethics Committee of the Faculty of Medicine of Vilnius University (150000-KT-346).

## 3. Results

### 3.1. Descriptive Statistics

The analysis of the data showed that students in the study had moderate levels of stress during the exam session and that higher levels of academic stress were associated with students’ workload and examinations (2.79 ± 0.60), and the lowest levels of stress related to academic expectations (3.21 ± 0.83) and academic self-perceptions (3.46 ± 0.50) (*p* < 0.001), which confirmed the first hypothesis (H1) (Table 2).

The responses of students from different years of study indicated that stress related to workload and examinations decreased with the number of years of study. Students in higher years of study experience less stress of this type than younger students. However, statistically significant differences were found only between second- and fourth-year students (Table 3).

The study revealed that stress related to academic self-perception tended to increase with years of study, but the differences between student groups were not statistically significant. However, stress related to academic expectations tended to increase—students in the 3rd and 4th years of study had higher stress related to academic expectations than students in the first year of study.

There was no difference in most motivation scale indicators between students in the different years. Significant differences were found in the analysis of the data on EM introjected regulation, which revealed a steady decline in this type of motivation from the first to the fourth year of study. In addition, students in the fourth year of the study had lower levels of IM to experience stimulation, but this difference was not statistically significant between years of study (Table 3). The study revealed a trend indicating that students in higher years of study tended to exhibit higher levels of amotivation; however, the differences between study years were not statistically significant.

### 3.2. Correlation Analysis

When analyzing the correlations between the stress subscales, a statistically significant relationship was found between academic expectations and academic self-perception (r = 0.485; p < 0.01), whereas no significant association was observed between academic expectations and stress related to workload and examinations (Table 4). Stress related to academic self-perception was significantly associated with all intrinsic and extrinsic motivation subscales (r = 0.170–0.344; p < 0.05), indicating that students who evaluated themselves more positively in academic terms also reported higher levels of motivation. In contrast, stress related to workload and examinations showed a statistically significant negative correlation with introjected regulation, a subtype of extrinsic motivation (r = −0.159; *p* < 0.01).

Amotivation had a statistically significant positive relationship with stress related to workload and examinations (r = 0.156; *p* < 0.05) and a significant negative association with academic self-perception (r = −0.268; *p* < 0.01). Additionally, the results also showed that both intrinsic and extrinsic motivation were significantly positively related to students’ subjective vitality (r = 0.194–0.356; *p* < 0.01). Notably, extrinsic forms of motivation, particularly introjected regulation, were more strongly correlated with vitality than intrinsic motivation. Thus, the study confirmed the second hypothesis (H2): students’ academic motivation and subjective vitality are significantly associated, although the strength of this relationship was below average.

However, no statistically significant correlations were found between any of the stress subscales and students’ vitality. Thus, the stress experienced by students during the examination period did not appear to have a clear impact on their subjective vitality levels. As a result, the third hypothesis (H3) was not supported in this study.

### 3.3. Multiple Regression Analysis

Given that the study showed that workload and examination-related stress were most pronounced in the participant group, we conducted regression analysis (Table 5). Multiple linear regression analysis revealed that the model explained 20.4% of the variance in students’ workloads and examination stress (R^2^ = 0.204). Significant predictors included age, course year, academic self-perception, and amotivation. Specifically, older students reported lower levels of stress (β = −0.115, *p* = 0.03), whereas those in higher years of study experienced higher stress (β = 0.131, *p* = 0.04). Additionally, students with higher academic self-perception reported higher levels of stress (β = 0.392, *p* < 0.001). Higher levels of amotivation were also associated with increased stress (β = 0.067, *p* = 0.044), but other predictors, such as autonomous and controlled motivation and vitality, were not significant.

## 4. Discussion

The uniqueness of this study lies in the methodological choice to analyze the psychological indicators of nursing study program students during the exam session, that is, during a period of intensive learning and increased academic stress. The students completed the questionnaires three days before each of the selected exams. This study analyzed the interrelationship between academic stress, academic motivation, and subjective vitality in an emotionally charged context; therefore, the collected data provide a unique opportunity to assess how academic tension and its challenges are reflected in students’ motivation and affect their vitality.

The study results showed that the participants had moderate academic stress, and students’ perceived academic stress was mainly related to workload and examinations. In addition, the study showed that older students noted that they experienced less workload and examinations related to stress than younger students. This finding is consistent with prior research indicating that academic stress tends to be higher among first-year students due to the demands of adaptation and lack of experience in managing study-related workloads [64,65]. Senior students often report lower stress levels as they acquire coping strategies and gain more autonomy in learning.

When analyzing the participants’ motivation, it was observed that the most expressed form of motivation was EM-identified regulation. This shows that students realize the personal importance of learning but are still strongly influenced by external expectations and social pressure, especially in the first year of study. Identified regulation reflects a more autonomous form of extrinsic motivation in which students internalize the value of academic tasks and perceive them as personally meaningful [66,67]. Although influenced by external factors, these students will likely be more engaged and persistent in their learning efforts.

When analyzing the differences in motivation between groups, a significant difference was found only in EM introjective regulation: first-year students had higher rates of introjective regulation than senior students. Introjective regulation refers to internal pressures, such as guilt, shame, or the need to prove worth. This often drives behavior, not out of genuine interest but out of self-imposed obligations [68]. The higher level among first-year students may reflect their greater sensitivity to academic expectations and their efforts to conform to perceived social or institutional standards.

It should be noted that the type of motivation IM to experience stimulation was the least pronounced. This finding aligns with prior studies showing that intrinsic motivation based on enjoyment and excitement tends to diminish under academic pressure, particularly during examination periods [69,70]. During periods of high stress, students often shift towards more externally regulated forms of motivation, limiting the expression of intrinsic motivation related to stimulation [66].

The subjective vitality levels of the study participants were moderate, and no statistically significant differences were observed between student groups across different years of study. The correlation analysis demonstrated that subjective vitality was significantly associated with intrinsic and extrinsic academic motivation forms. On the one hand, these findings support the theoretical assumption that psychological energy and vitality stem from an authentic, internal source of motivation, as posited by self-determination theory [53]. Specifically, intrinsic motivation—particularly to know and accomplish—has been consistently linked to higher vitality, well-being, and sustained engagement [71,72].

However, research has shown that external forms of regulation, especially introjected regulation, correlate with students’ vitality. Such motivation is related to external pressures, such as guilt, anxiety, or the need to validate self-worth [73,74]. Although this regulation does not reflect genuine autonomy, in certain situations, such as exam periods, it can rarely energize students and potentially contribute to increased vitality despite its emotionally taxing nature.

Although no statistically significant relationships were found in the analysis of the relationship between vitality and academic stress, the established relationships between vitality and forms of extrinsic motivation during the exam session allow us to hypothesize an internal conflict between duty and intrinsic desire to explain how perceived stress can affect vitality through motivational regulation. In other words, increased academic demands promote intrajectional regulation, i.e., internalized pressure that temporarily increases subjective vitality by mobilizing psychological resources. Such a possible mediating role of controlled motivation highlights the complexity of students’ adaptation during stressful academic periods.

Our research findings, consistent with those of other authors [64], indicate that stress related to workload and examinations constitutes one of the primary forms of stress experienced by students. However, the results of regression analysis showed that the variables included in the study, such as motivation and vitality, explained only 20% of the variance in stress associated with workloads and examinations. This suggests that other psychological and social factors play a significant role in the stress experienced by students during exam sessions. According to recent research [75], weak social support and poor interpersonal relationships increase stress levels because students feel isolated and insecure.

Students’ academic self-perceptions have a significant impact on their engagement in the study process. However, it is important to note that academic self-perception may be associated with better academic performance as well as increased expectations and self-critical pressure during exams, thus indirectly increasing stress intensity [64,76]. Thus, academic stress is associated with anxiety [77] and higher levels of procrastination [78]. Therefore, insufficient time management and the undeveloped self-regulation skills of students further increase academic stress in students [79]. Therefore, the complexity of the factors causing students’ stress during exams highlights the need for comprehensive research that analyzes the interaction between various factors.

### 4.1. Study Limitations

The present study has some limitations:

- First, it used a single university sample, making it difficult to generalize the results to a wider student population.
- Second, data were collected using self-reporting questionnaires. No objective psychological measures were used, which may have affected the reliability of the data, owing to the potential subjectivity of the responses.
- Third, the study was conducted cross-sectionally during the examination period, which is typically characterized by increased learning intensity and stress levels. However, in developing research in this area, it would be useful to conduct comparative studies that analyze the stress experienced by students during different periods of study. Furthermore, from the perspective of the changing learning paradigm, qualitative research could provide valuable information that would not only assess but also explain and better understand the experience of stressful situations from the students’ perspective. This is especially important as the educational paradigm shifts from a directive style to a teacher–student “co-creation” approach.
- Fourth, the study did not collect information about the social environment and lifestyle characteristics of the students, which could significantly expand the understanding of the stress experienced by students during the session. Therefore, the analysis of academic stress as a phenomenon requires a holistic approach that includes not only individual psychological factors but also institutional and social aspects of the environment.

### 4.2. Applicability of Research Results

Despite the limitations, the results of this study are important for understanding issues related to the training of future nursing professionals. This study revealed the impact of the educational environment on the academic stress and motivation of nursing students. This emphasizes the need to review the principles of organizing the educational process to reduce excessive stress related to educational factors. It is important to create an environment for assessing student learning, knowledge, and skills to promote both extrinsic and intrinsic motivation. Thus, the results of this study contribute to the implementation of long-term goals of health sciences, as motivated and psychologically strong nursing students will become competent and empathetic healthcare professionals in the future.

## 5. Conclusions

According to the academic stress scale, nursing students experience average academic stress during the exam session, with the highest being academic stress associated with workload and examinations. Academic stress related to workload and exams is determined by both demographic factors, such as age and year of study, and psychological factors, including academic self-perception and amotivation, highlighting the multifaceted nature of stress experienced by nursing students.

Students’ academic motivation was characterized by high extrinsic motivation—identified regulation and intrinsic motivation—to know. A significant difference in motivation between student groups was found in the introjected regulation of extrinsic motivation, and the study showed a tendency for this motivation to decrease with the years of study.

Students’ subjective vitality was at an average, but no statistically significant correlation was found between students’ academic stress and subjective vitality. However, the study showed that students’ subjective vitality is related to intrinsic motivation—to know and accomplish things—and all forms of extrinsic motivation. Thus, external forms of regulation, especially intrapersonal regulation, were also significantly related to students’ subjective vitality. This means that, under unusual conditions, that is, during exam sessions, external regulation can stimulate an increase in students’ vitality by mobilizing their energy.

## Figures and Tables

**Table 1:** Distribution of student nurses by course of study.

Year of Study	*n*	Proc
1	43	22.0
2	49	26.1
3	50	26.6
4	46	24.5

**Table 2:** Indicators of nursing students’ perceptions of academic stress.

Subscales	Rating *	Mean (SD)	F-Value	*p*-Value
Academic expectations	1–5	3.21 (0.83)	45.46	*p* < 0.001
Workload and examinations	1–5	2.79 (0.60)		
Academic self-perceptions	1–5	3.46 (0.50)		

Note: * Lower value indicates higher level of stress.

**Table 3:** Data on academic stress, academic motivation, and vitality of nursing students.

Subscales	Rating	Student Groups				χ^2^	*p*-Value **
		1 Year of Study	2 Years of Study	3 Years of Study	4 Years of Study		
1. Academic expectations	1–5 *	3.25 (0.92)	3.40 (0.76)	3.03 (0.82)	3.15 (0.78)	38.50	*p* = 0.01
2. Workload and examinations	1–5 *	2.81 (0.55)	2.66 (0.53)	2.80 (0.53)	2.90 (0.60)	64.81	*p* = 0.02
3. Academic self-perceptions	1–5 *	3.40 (0.43)	3.40 (0.47)	3.49 (0.48)	3.51 (0.55)	40.39	*p* > 0.05
4. IM to know	1–7	5.61 (1.32)	5.54 (1.49)	5.64 (1.28)	5.70 (1.47)	43.61	*p* > 0.05
5. IM to accomplish things	1–7	5.25 (1.43)	5.05 (1.55)	5.20 (1.25)	5.20 (1.56)	42.77	*p* > 0.05
6. IM to experience stimulation	1–7	4.78 (1.29)	4.67 (1.73)	4.76 (1.38)	4.23 (1.91)	59.40	*p* > 0.05
7. EM identified regulation	1–7	5.93 (1.20)	5.70 (1.44)	5.84 (1.20)	5.80 (1.39)	45.05	*p* > 0.05
8. EM introjected regulation	1–7	5.33 (1.26)	5.07 (1.48)	4.82 (1.48)	4.79 (1.62)	98.71	*p* = 0.03 *p* = 0.04
9. EM external regulation	1–7	5.36 (1.19)	5.09 (1.47)	5.43 (1.39)	5.32 (1.57)	44.12	*p* > 0.05
10. Amotivation	1–7	2.02 (1.21)	2.14 (1.25)	2.34 (1.57)	2.37 (1.54)	56.13	*p* > 0.05
11. Vitality	1–7	4.53 (1.24)	4.39 (1.32)	4.10 (1.23)	4.22 (1.36)	108.79	*p* > 0.05

Note: * Lower value indicates higher level of stress. ** Only statistically significant differences between groups are shown in the table.

**Table 4:** Correlation data for stress, motivation, and vitality variables in nursing students.

Subscales	1	2	3	4	5	6	7	8	9	10	11
1. Academic expectations		0.082	0.485 **	0.096	0.170 *	0.317 **	0.041	0.214 **	0.016	−0.143	0.113
2. Workload and examinations			0.267 **	−0.085	−0.142	−0.121	−0.055	−0.159 *	−0.073	0.156 *	−0.134
3. Academic self-perceptions				0.307 **	0.344 **	0.363 **	0.229 **	0.170 *	0.203 **	−0.268 **	0.073
4. IM to know					0.822 **	0.580 *	0.860 **	0.487 **	0.686 **	−0.534 **	0.245 **
5. IM to accomplish things						0.671 **	0.747 **	0.564 **	0.629 **	−0.446 **	0.194 **
6. IM to experience stimulation							0.519 **	0.478 **	0.548 **	−0.446 **	0.069
7. EM identified regulation								0.496 **	0.724 **	−0.590 **	0.246 **
8. EM introjected regulation									0.556 **	−0.273 **	0.356 **
9. EM external regulation										−0.410 **	0.254 **
10. Amotivation											−0.095
11. Vitality											

Note: red color indicates a positive, while blue color indicates a negative correlation between variables. * *p* < 0.05; ** *p* < 0.01.

**Table 5:** Multiple linear regression of workload and examination stress.

Predictors	B	Sb	Beta	t	*p*	95% CI for B
Age	−0.115	0.053	−0.249	−2.172	0.03	[−0.220, −0.011]
Gender	−0.001	0.001	−0.089	−1.270	0.21	[−0.001, 0.001]
Course year	0.131	0.063	0.240	2.068	0.04	[0.006, 0.256]
Autonomous motivation	−0.032	0.0538	−0.068	−0.595	0.55	[−0.138, 0.074]
Controlled motivation	−0.037	0.0501	−0.080	−0.745	0.46	[−0.136, 0.062]
Amotivation	0.066	0.0328	0.156	2.025	0.04	[0.001, 0.131]
Vitality	−0.053	0.0342	−0.114	−1.544	0.12	[−0.120, 0.015]
Academic expectations	0.028	0.0567	0.039	0.490	0.63	[−0.084, 0.140]
Academic self-perceptions	0.392	0.0835	0.385	4.692	<0.001	[0.227, 0.556]

Adjusted R^2^ = 0.164, F(9, 178) = 5.08, *p* < 0.001.

## Data Availability

The data presented in this study are available on request from the corresponding author due to confidentiality agreements with participants.

## Public Involvement Statement

No public involvement in any aspect of this research.

## Guidelines and Standards Statement

This manuscript was drafted against the STROBE (Strengthening the Reporting of Observational Studies in Epidemiology) guidelines for cross-sectional observational research. The checklist was used to ensure comprehensive and transparent reporting.
